# Production of Pumilarin and a Novel Circular Bacteriocin, Altitudin A, by *Bacillus altitudinis* ECC22, a Soil-Derived Bacteriocin Producer

**DOI:** 10.3390/ijms25042020

**Published:** 2024-02-07

**Authors:** Irene Lafuente, Ester Sevillano, Nuria Peña, Alicia Cuartero, Pablo E. Hernández, Luis M. Cintas, Estefanía Muñoz-Atienza, Juan Borrero

**Affiliations:** 1 Departamento de Nutrición y Ciencia de los Alimentos (NUTRYCIAL), Sección Departamental de Nutrición y Ciencia de los Alimentos (SD-NUTRYCIAL), Facultad de Veterinaria, Universidad Complutense de Madrid (UCM), Avenida Puerta de Hierro, s/n, 28040 Madrid, Madrid, Spain; irelafue@ucm.es (I.L.); estsev01@ucm.es (E.S.); nuriapen@ucm.es (N.P.); ehernan@ucm.es (P.E.H.); lcintas@ucm.es (L.M.C.); 2 Centro de Educación Infantil, Primaria y Secundaria Obligatoria (CEIPSO) El Cantizal, Avenida Atenas s/n, 28232 Las Rozas, Madrid, Spain; acuarterovillalvilla@educa.madrid.org

**Keywords:** circular bacteriocins, antimicrobial peptides, *Bacillus altitudinis*, in vitro cell-free protein synthesis (IV-CFPS), split-intein-mediated ligation (SIML) of peptides

## Abstract

The rise of antimicrobial resistance poses a significant global health threat, necessitating urgent efforts to identify novel antimicrobial agents. In this study, we undertook a thorough screening of soil-derived bacterial isolates to identify candidates showing antimicrobial activity against Gram-positive bacteria. A highly active antagonistic isolate was initially identified as *Bacillus altitudinis* ECC22, being further subjected to whole genome sequencing. A bioinformatic analysis of the *B. altitudinis* ECC22 genome revealed the presence of two gene clusters responsible for synthesizing two circular bacteriocins: pumilarin and a novel circular bacteriocin named altitudin A, alongside a closticin 574-like bacteriocin (CLB) structural gene. The synthesis and antimicrobial activity of the bacteriocins, pumilarin and altitudin A, were evaluated and validated using an in vitro cell-free protein synthesis (IV-CFPS) protocol coupled to a split-intein-mediated ligation procedure, as well as through their in vivo production by recombinant *E. coli* cells. However, the IV-CFPS of CLB showed no antimicrobial activity against the bacterial indicators tested. The purification of the bacteriocins produced by *B. altitudinis* ECC22, and their evaluation by MALDI-TOF MS analysis and LC-MS/MS-derived targeted proteomics identification combined with massive peptide analysis, confirmed the production and circular conformation of pumilarin and altitudin A. Both bacteriocins exhibited a spectrum of activity primarily directed against other *Bacillus* spp. strains. Structural three-dimensional predictions revealed that pumilarin and altitudin A may adopt a circular conformation with five- and four-α-helices, respectively.

## 1. Introduction

Antibiotics have undeniably revolutionized modern medicine, saving countless lives by effectively combatting bacterial infections. However, their overuse, misuse, and inadequate regulation have given rise to a growing global health crisis due to bacterial antimicrobial resistance (AMR). AMR occurs when bacteria develop the ability to withstand the effects of antibiotics, rendering these compounds progressively ineffective [1]. The World Health Organization (WHO) has issued a severe warning that, if current activity continues, AMR is projected to become the leading cause of human deaths by 2050, surpassing even cancer and other major diseases [2,3,4]. This worrisome situation underscores the urgent need for public awareness of the responsible use of antibiotics and the discovery and development of novel antimicrobial compounds, from both known and untapped sources, not only to combat existing drug-resistant infections, but also to prepare for the unforeseen microbial threats of the future [1,5].

Many antibiotics used in clinical applications, such as streptomycin, vancomycin or tetracycline, have been identified from bacteria isolated from soil [6,7]. However, in recent years, the identification of new antibiotics has declined dramatically with a few scarce exceptions, such as the discovery of teixobactin and clovibactin, produced by uncultivable soil-derived bacteria [7,8]. Although the identification of new antibiotics has been significantly reduced, it is known that soil-derived bacteria, in addition to antibiotics, have the potential to produce other antimicrobial compounds [9,10,11], such as bacteriocins and other secondary metabolites, which are being assessed for their potential clinical application as viable alternatives to traditional antibiotics [12,13].

Bacteriocins are ribosomally synthesized antimicrobial peptides produced by bacteria, being non-toxic to eukaryotic cells but with a strong antimicrobial activity, at the nM range, against pathogens including antibiotic multiresistant or extremely resistant bacterial strains [14,15]. Most bacteriocins are synthesized as biologically inactive precursors or prepeptides, containing an N-terminal extension that is cleaved off during export to generate their biologically active or mature form. The mature bacteriocin peptides were initially classified into two main classes: class I or lantibiotics, with lanthionine-containing post-translationally modified amino acid residues, and class II bacteriocins with unmodified amino acid residues [16]. However, currently, the class I group of modified bacteriocins includes all ribosomally synthesized and post-translationally modified peptides (RiPPS) that undergo enzymatic modification during biosynthesis (e.g., lanthipeptides, thiopeptides, lasso peptides, circular and cyclized peptides, sactipeptides and others), whereas the class II bacteriocins groups peptides that do not require posttranslational modification enzymes for their maturation [12,17,18].

An interesting group within the class I bacteriocins is the one comprising the head-to-tail cyclized antimicrobial peptides, commonly referred to as circular bacteriocins [19,20,21,22]. Circular bacteriocins are synthesized as linear precursor peptides, containing a leader peptide (2 to 48 amino acid residues long) which is cleaved off during the maturation process. The linear peptides are cyclized by the formation of an amide bond between the N-and C-terminal residues, before being exported out of the cell. The synthesis and secretion of circular bacteriocins involves proteins encoded by genes that are usually clustered together, including a minimum of five to seven genes encoding the bacteriocin precursor peptide, a stage II sporulation protein M (SpoIIM) previously referred as a protein of the DUF95 superfamily of proteins, an ATP-binding protein, and one or more proteins of yet unknown functions. Although the detailed mechanism by which these bacteriocins circularize has yet to be determined, most studies suggest that leader peptide removal and cyclization are two separate processes [23,24,25]. Furthermore, their circular conformation gives cyclized peptides an increased thermo-, pH- and proteolytic stability compared to their linear counterparts. These features make circular bacteriocins highly promising antimicrobial peptide candidates for human, animal and industrial applications [18,25,26].

Here we report the identification, biochemical and genetic characterization of a novel head-to-tail circular bacteriocin altitudin A, produced by *Bacillus altitudinis* ECC22 isolated from a screen of soil-derived bacteria with antimicrobial activity, by high school students involved in the Service-Learning and Citizen-Science Project MicroMundo [27]. A genome-based comprehensive bioinformatic analysis of *B. altitudinis* ECC22 and the evaluation of the synthesis, expression and antimicrobial activity of the putative circular bacteriocins, encoded by this strain, was evaluated through an in vitro cell-free protein synthesis (IV-CFPS) protocol coupled to a split-intein-mediated ligation (SIML) procedure, and by their in vivo production by recombinant *E. coli* cells. The biochemical, genetic and functional characterization of the bacteriocins encoded by *B. altitudinis* ECC22 confirmed the coproduction by this strain of pumilarin, a circular bacteriocin previously characterized [26], and a novel bacteriocin, termed altitudin A. Both bacteriocins showed antagonistic activity against Gram-positive strains, primarily *Bacillus* spp. and *Listeria* spp. To our knowledge, this is the first report of the simultaneous production of two circular bacteriocins by a single bacterial isolate. Understanding the vast and diverse antimicrobial potential of soil-derived microorganisms also offers a promising opportunity for the identification of new antimicrobial compounds, different from antibiotics, renewing hope in the ongoing fight against bacterial antibiotic resistance.

## 2. Results

### 2.1. Isolation of Bacillus altitudinis ECC22 from Soil-Derived Samples

A number of bacterial colonies grown in TSB agar plates and isolated from soil-derived samples were screened for their direct antimicrobial activity against the Gram-positive indicator *Kocuria rhizophila* CECT 241 and the Gram-negative bacteria *Acinetobacter baylyi* ATCC 33305 (Figure S1). While none of the isolates displayed inhibitory activity against *A. baylyi* ATCC 33305, 21 isolates showed antagonistic activity against *K. rhizophila* CECT 241. One of the isolates showing the largest antimicrobial activity (isolate 13, Figure S2), was selected for further evaluation. The PCR-specific amplification and sequencing of the 16S rRNA gene of the purified isolate allowed its taxonomic identification as *Bacillus altitudinis*. Following the Micromundo nomenclature, this isolate was named *B. altitudinis* ECC22. The letters EC stands for El Cantizal, C stands for Carlota (name of the student who isolated the strain), and 22 stands for the year in which the isolation took place.

### 2.2. Identification of Bacteriocin Gene Clusters in the B. altitudinis ECC22 Genome

**Figure 1:**
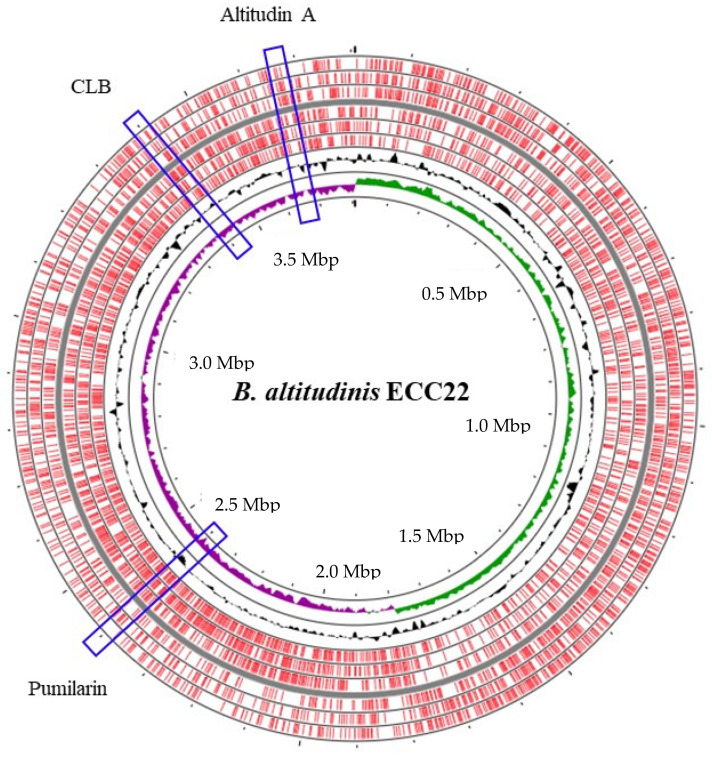
*Bacillus altitudinis* ECC22 genome map generated using the CGView server. The red squares represent the coding sequences (CDS). The black plot shows the GC content, the green plot shows the CG skew +, and the purple plot shows the CG skew −. The position of the gene clusters for pumilarin, altitudin A, and the closticin 574-like bacteriocin (CLB) are highlighted with a blue rectangle.

The hybrid assembly of the genome sequence of *B. altitudinis* ECC22 with the Illumina and Oxford Nanopore Technologies (ONT) confirmed a 3,807,059-bp circular chromosome with a 41.3% G + C content (Table S1) and 3,929 protein-coding genes (GenBank accession number CP137888). No plasmids were identified. The species prediction made by the SpeciesFinder and KmerFinder web servers confirmed the identification of the strain as *B. altitudinis*. The subsequent analysis of the *B. altitudinis* ECC22 genome with the bacteriocin mining tools BAGEL v.4.0 and antiSMASH revealed the identification of at least three different bacteriocin gene clusters located in separated sites within its chromosome (Figure 1).

**Figure 2:**
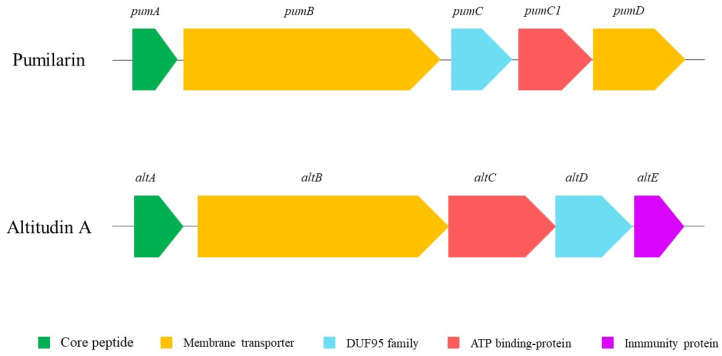
Genetic organization of the pumilarin and altitudin A gene clusters in the *B. altitudinis* ECC22 genome.

**Figure 3:**
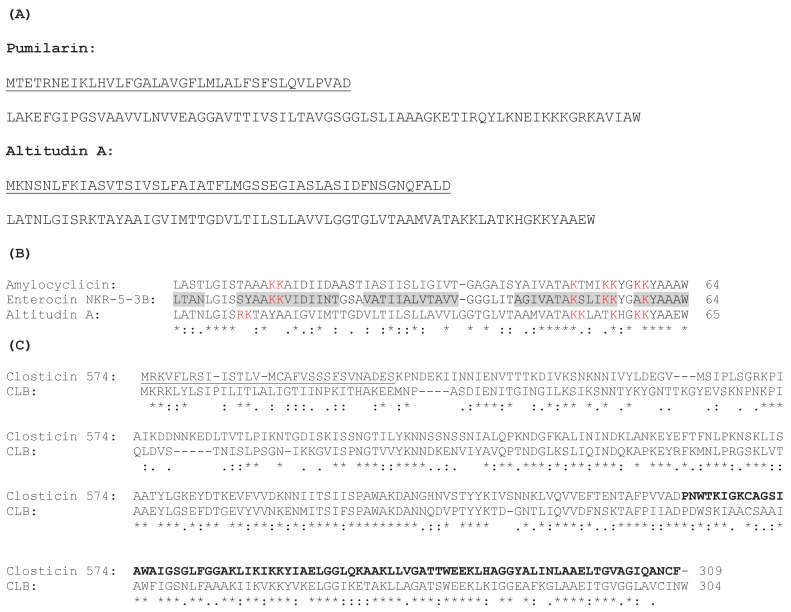
(**A**) Amino acid sequences of the pumilarin and altitudin A prepeptides, with the leader sequences underlined and separated from the mature peptides. (**B**) Multiple-sequence alignment of altitudin A with the circular bacteriocins amylocyclicin and enterocin NKR-5-α. The dark grey highlighting shows the allocation of experimentally confirmed α-helices. The cationic amino acid residues are in red. (**C**) Amino acid sequence alignment of the bacteriocin closticin 574 with the predicted closticin 574-like peptide (CLB). The leader sequence is underlined, and the mature peptide is in bold. An asterisk (*) indicates a single fully conserved residue, a colon (:) indicates conservation within groups of residues with strongly similar properties, and a period (.) indicates conservation within groups of residues with weakly similar properties.

One gene cluster encodes the bacteriocin pumilarin, previously characterized as a circular bacteriocin, produced by *Bacillus pumilus* B4107 [26]. The second identified gene cluster contains a set of genes presumably responsible for the synthesis and production of a putative circular bacteriocin, termed altitudin A (Figure 2). The altitudin A gene cluster is 4,475 bp long, and the organization of the genes within the cluster is similar to other circular bacteriocins [28]. The mature altitudin A has a 46% identity with amylocyclin A [29] and a 52% identity with enterocin NKR-5-3B [30]. Based on this homology, it could be hypothesized that the structural *alt* gene encodes a 114-amino acid precursor with a 49-amino acid leader sequence which, once processed, results in a circular bacteriocin of 65-amino acids, finally termed altitudin A (Figure 3). The third bacteriocin gene cluster identified in the *B. altitudinis* ECC22 genome encodes a putative bacteriocin with a 50% identity to closticin 574 (Figure 3), an unusual class II 82-amino acid bacteriocin characterized in *Clostridium tyrobutyricum* ADRIAT 932 [31]. 

### 2.3. In Vitro Cell-Free Protein Synthesis (IV-CFPS) Protocol Coupled to a Split-Intein-Mediated Ligation (SIML) Procedure for the Synthesis, Production and Determination of the Antimicrobial Activity of Pumilarin and Altitudin A

**Figure 4:**
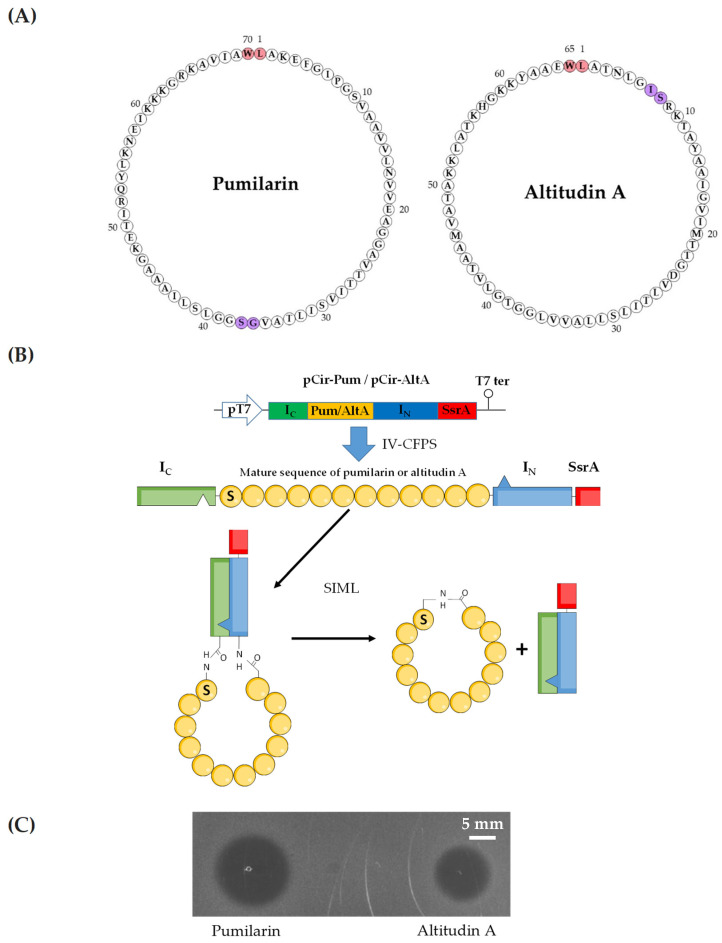
Bacteriocin circularization by the IV-CFPS protocol coupled to the SIML procedure. (**A**) Amino acid sequence of mature pumilarin and altitudin A. In red, the amino acids implicated in the head-to-tail circularization of native pumilarin and altitudin A. In blue, the amino acids selected for the IV-CFPS coupled to the SIML procedure, and the in vivo production of pumilarin and altitudin A. (**B**) Schematic representation of the pUC-derived expression vectors encoding the gene products of interest, the recombinant peptides produced by IV-CFPS, and the SIML procedure where the Npu DnaE intein fragments, I_C_ and I_N_, interact to form an active peptide that splices, including circularization of the mature peptides. (**C**) Antimicrobial activity against *Pediococcus damnosus* CECT 4797 by a spot-on-agar test (SOAT) using 5 µL fractions of pumilarin and altitudin A, produced individually by the IV-CFPS/SIML method.

To assess the synthesis, production and antimicrobial activity of pumilarin and the putative circular bacteriocin altitudin A, two synthetic gene constructs containing the C- and N-terminal fragments from the *Nostoc puntiforme* (Npu) DnaE split-intein, flanking the genes coding the mature peptide of both bacteriocins, were designed. For pumilarin, S37 was selected as the first amino acid residue in the linear conformation, and G36 as the last residue. For altitudin A, S8 was selected as the first amino acid residue in the linear conformation, and I7 as the last residue. In addition, a protein degradation tag (SsrA) was included in the C-terminus of the gene constructs. The insertion of the gene constructs under the control of a pUC-derived expression vector permitted completion of the plasmids pCir-Pum, encoding pumilarin, and pCir-AltA, encoding altitudin A (Figure 4). Both plasmids were used as templates for the in vitro cell-free protein synthesis (IV-CFPS) coupled to the split-intein-mediated ligation (SIML) of bacteriocins previously developed by our research group [18]. Importantly, the pumilarin and altitudin A produced by the IV-CFPS and SIML procedure showed antimicrobial activity against *P. damnosus* CECT4797 as the indicator microorganism, thus confirming their biological functionality (Figure 4).

However, the IV-CFPS synthesis of the PCR-amplified closticin 574-like bacteriocin gene (*clb*), did not show antimicrobial activity against *P. damnosus* 4797 and *C. perfringens* DICM15/00067-5A as the indicator microorganisms, thus suggesting that either the indicators used in this study were not sensitive to the bacteriocin CLB, or that the structural gene encodes a non-functional bacteriocin or a protein with a different functionality.

### 2.4. Purification of the Pumilarin and Altitudin A Produced by B. altitudinis ECC22, and MALDI-TOF MS Analysis and LC-MS/MS-Derived Targeted Proteomics Identification, Combined with Massive Peptide Analysis of the Purified Bacteriocins

**Figure 5:**
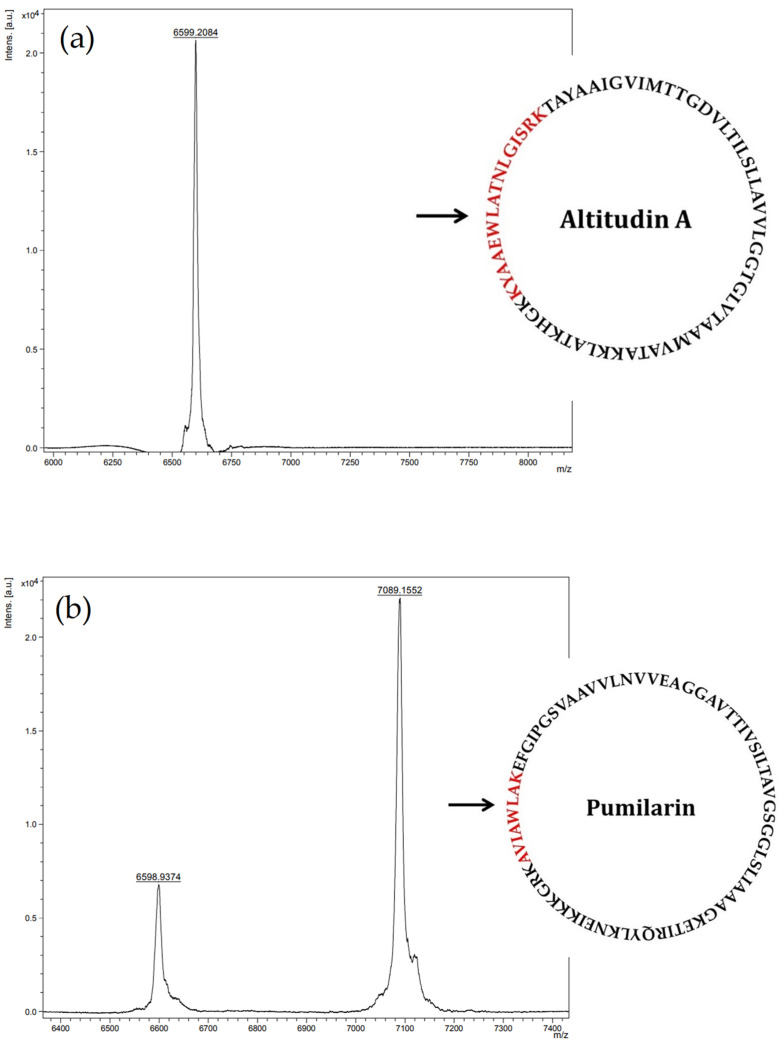
MALDI-TOF MS analysis of fractions 12 (**a**) and 15 (**b**) after the second RP-HPLC round of purification of the CFS of *B. altitudinis* ECC22. The peptides with a molecular mass (*m*/*z*) of 6598.93 and 7089.15 are for altitudin A and pumilarin, respectively. The full amino acidic sequences of altitudin A and pumilarin are in the right side of the figures. In red, the sequences containing the circularization site, identified by LC-MS/MS after digestion of fractions 12 and 15. The black arrow shows the head-to-tail circularization of the native bacteriocins.

We attempted the purification of the antimicrobial compounds in the supernatants (CFS) of *B. altitudinis* ECC22 to confirm the synthesis, production and secretion of pumilarin and altitudin A. The CFS of *B. altitudinis* ECC22 were subjected to precipitation with ammonium sulphate, desalted by gel filtration, and further subjected to hydrophobic-interaction chromatography, followed by two rounds of reverse-phase chromatography in an ÄKTA RP-FPLC system. After the second round of RP-FPLC, a measurable antimicrobial activity was observed in two separated fractions, eluted at 55% (Fraction 12) and 66% (Fraction 15) isopropanol with 0.1% (*v*/*v*) trifluoroacetic acid (TFA) (Figure S3). Analysis of the eluted fraction 12 by MALDI-TOF MS revealed the presence of a single peptide peak with a molecular mass of 6599.20 Da, while the analysis of the eluted fraction 15 revealed the presence of two independent peptide peaks with a molecular mass of 6598.93 Da and 7089,15 Da (Figure 5). The 18 Da difference between the deduced (6615.93 Da) and the observed (6598.93 Da) molecular mass of the first identified peptide (Figure 5a) is compatible with a dehydration during the formation of an amide bond between the N- and C-terminal residues of the altitudin A. The difference between the observed (7089.15 Da) and the deduced (7105.42 Da) molecular mass of the second identified peptide (Figure 5b) is also compatible with the loss of 18 Da during the circularization of pumilarin.

To confirm the presence of altitudin A and pumilarin and their circular structure in the supernatants of *B. altitudinis* ECC22, the RP-FPLC-eluted fractions with antimicrobial activity were subjected to trypsin digestion and an LC-MS/MS analysis of the resulting peptides, for the determination of its molecular mass and amino acid sequence, using a targeted proteomics approach combined with a massive peptide analysis. Among the peptide fragments identified in the RP-FPLC-purified fraction 12, most of them were compatible with the amino acid sequence of altitudin A, but one of them (YLNEIK) was compatible with the sequence of pumilarin. These results confirmed that the purification of both bacteriocins to homogeneity was not achieved (Table 1). However, the identification in both purified fractions of peptide fragments containing the amino acids WL linked together, such as in fragment AVIAWLAK for pumilarin and in fragment YAAEWLATNLGISRK for altitudin A, confirmed their native head-to-tail covalent link in the purified supernatants of *B. altitudinis* ECC22 (Table 1). 

### 2.5. In Vivo Production and Antimicrobial Activity of Pumilarin and Altitudin A, Produced by Recombinant E. coli BL21 (DE3) Cells

Since pumilarin and altitudin A were not purified to homogeneity from the CFS of *B. altitudinis* EC22, they were produced in vivo by recombinant *E. coli* BL21 (DE3) (pCir-Pum) and *E. coli* BL21 (DE3) (pCir-AltA), producers of pumilarin and altitudin A, respectively. The cellular soluble fraction (CSF) from the sonicated *E. coli*-producer cells was subjected to precipitation with ammonium sulphate, purified by hydrophobic-interaction chromatography, and the presence of pumilarin and altitudin A in the purified fractions was confirmed by MALDI-TOF MS. The antimicrobial activity of pumilarin and altitudin A in the purified fractions was evaluated for both bacteriocins, alone or combined. Both bacteriocins showed antimicrobial activity against most of the *Bacillus* spp., *Listeria seeligeri* CECT 917 and *L. monocytogenes* CECT 4032 strains tested. Pumilarin was also active against *Lactococcus lactis* LMG3797, *L. lactis* LMG3798 and *Streptococcus suis* CECT 958. No clear synergistic effect was observed when both bacteriocins were tested together (Table 2).

### 2.6. Three-Dimensional (3D) Structures of Pumilarin and Altitudin A, Predicted by Homology Modelling

**Figure 6:**
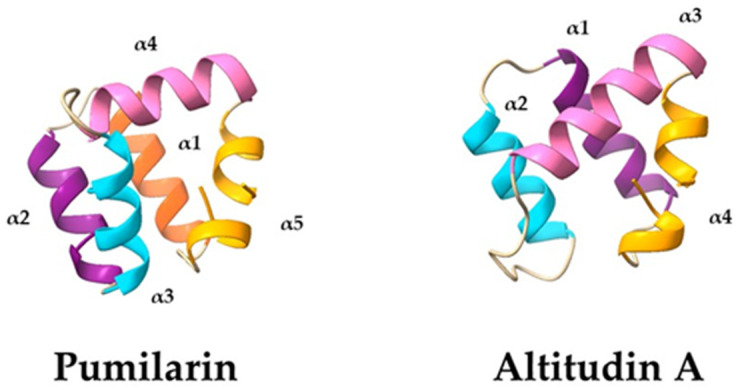
Three-dimensional (3D) structural model of pumilarin and altitudin A, created by RobettaFold. Each α-helix is numerated and shows a different color.

Good-quality 3D structures were predicted for the circular bacteriocins pumilarin and altitudin A. According to the models generated by the Robetta server, pumilarin contains five alpha-helices (Figure 6), which is in agreement with previous observations suggesting a similar structure and charge distribution between pumilarin and enterocin AS-48 [26]. On the other hand, the model generated for altitudin A suggests that the bacteriocin contains four alpha-helices, i.e., R9-T21 (α1), V25-V35 (α2), A44-K56 (α3) and K59-N4 (α4), and the head-to-tail circularization occurs within the α4 between residues L1 and W65. These four alpha-helices are connected by loops of two to eight residues with different glycine residues (G6, G23, G37, G38, G40 and G58) which are known to provide a conformational flexibility to these loops [32]. This structure is similar to that of enterocin NKR-5-3B, whose 3D conformation has been solved experimentally [30].

## 3. Discussion

Antimicrobial resistance has become a serious global health threat. Thus, there is an urgent need to raise awareness among health professionals, politicians, and citizens on the responsible use of antibiotics, while involving young researchers and students in the search for short-term solutions [33]. The Spanish Service-Learning Project Micromundo (https://www.ucm.es/small-world-initiative/proyecto, accessed on 8 January 2024) has the aim of conveying this message from the Universities to the schools, involving Spanish high-school students in an international project (Small World Initiative and Tiny Earth Projects) where participants from all over the world screen, every year, thousands of bacteria obtained from soil-derived samples in order to identify novel antimicrobials (https://www.smallworldinitiative.org/; https://tinyearth.wisc.edu/, accessed on 8 January 2024).

In 2022, students from the CEIPSO el Cantizal participating in the Micromundo project, isolated, from soil-derived samples, a bacterial strain with high antimicrobial activity against a sensitive bacterial indicator. The strain was taxonomically identified as *B. altitudinis* ECC22. *Bacillus* is among the major bacterial genus found in soil, and is recognized for producing valuable antibiotics and other antimicrobial compounds, including circular bacteriocins such as amylocyclicin, produced by *Bacillus amyloliquefaciens* [29], and pumilarin, produced by *B. pumilus* B4107 [26], but also other non-ribosomally synthesized peptides (NRPs) and polyketides (PKs) [11,34]. Recently, different works mining the diversity of circular bacteriocins in sequenced microbial genomes have identified *Bacillus* spp. as the predominant bacteria among those encoding putative circular bacteriocins [26,28,35,36]. The circular peptide backbone of these bacteriocins contributes to their remarkable stability to proteolytic resistance and structural integrity under a wide temperature and pH range [32]. Perhaps the production of circular bacteriocins by *Bacillus* spp. may be associated with the need for successful competition with other bacteria in a complex ecological niche, such as the soil. 

The hybrid assembly of the genome of *B. altitudinis* ECC22 confirmed a circular chromosome with no plasmids (Figure 1), and the identification, using the BAGEL v.4.0 and antiSMASH servers, of at least three putative biosynthetic bacteriocin gene clusters. The first cluster in the *B. altitudinis* ECC22 genome is associated with the synthesis and production of pumilarin, a 70 amino acid circular bacteriocin. This cluster is highly similar to that described in *Bacillus pumilus* B4107 for genes involved in the synthesis (*pumA*), production (*pumB* and *pumC*) and transport (*pumC1* and *pumD*) of pumilarin [26]. This bacteriocin gene cluster lacks a specific immunity-related gene (Figure 2) which has also been observed in other circular bacteriocin clusters recently identified by the in silico mining of published bacterial genomes [28,35]. However, the presence in the gene cluster of *B. altitudinis* ECC22 of the gene *pumC1* encoding the sporulation protein M (SpoIIM), also recognized as DUF95, and the *pumB* and *pumC1* genes encoding a putative membrane transporter and an ATP-binding protein, suggests that the SpoIIM/DUF95 superfamily protein may have a dual function in the synthesis of pumilarin, such as an immunity-associated transporter and a secretion-aiding agent, and as the role suggested for the DUF95 protein in the synthesis, secretion and immunity of the circular leucocyclin Q, produced by *Leuconostoc mesenteroides* TK41401 [24]. 

The second bacteriocin gene cluster identified in *B. altitudinis* EC222 has been observed in different *Bacillus* spp., including *B. pumilus* (AHL73410.1 and OBS83806.1), *B. altitudinis* (OJT66332.1, OPW96241.1 and PGD46397.1), *B. stratosphericus* (OQP18696.1) and *B. safensis* (OUK96109.1), during the mining for circular bacteriocins in sequenced microbial genomes [28]. However, the synthesis, production, secretion and antimicrobial activity of the putative circular bacteriocin precursors has not yet been determined. Thus, based on the homologies of the bacteriocin gene cluster of *B. altitudinis* ECC22 with that observed for the synthesis of the enterocin NKR-5-3B, produced by *E. faecium* NKR-5-3 [37], the gene (*altA*) encoding the putative circular bacteriocin named altitudin A was predicted to encode a 114-amino acid prepeptide, including a 49-amino acid leader peptide and a 65-amino acid propeptide domain that, upon the cleavage of the leader sequence and a head-to-tail ligation between residues L1 and W65, gives the active circular bacteriocin (Figure 3). Other genes in the bacteriocin-related cluster were predicted to encode a membrane protein (*altB*), an ABC-binding protein (*altC*), a protein with the SpoIIM/DUF95 motif (*altD*) and an immunity protein (*altE*) (Figure 2). 

The third bacteriocin-related cluster in the *B. altitudinis* ECC22 genome holds a gene encoding a peptide with a 50% similarity to the bacteriocin closticin 574, produced by *Clostridium tyrobutyricum* ADRIAT 932 [31]. Closticin 574 is produced as a 309 amino acid pre-protein that, after secretion and processing, gives rise to an antimicrobial peptide of 82 amino acids. The first 27 amino acids of the full-length protein probably constitute a general signal peptide, while the secreted protein is most probably further processed extracellularly either by a general or by a specific proteinase [31]. By comparing the amino acid sequence of closticin 574 with that of the putative bacteriocin encoded by *B. altitudinis* ECC22, we could predict a tentatively termed bacteriocin CLB (Closticin-Like-Bacteriocin), with the mature bacteriocin being 83 amino acids long (Figure 3).

To confirm that the antimicrobial activity of *B. altitudinis* ECC22 could be attributed to the synthesis and production of the encoded bacteriocins pumilarin, altitudin A and CLB, the bacteriocins pumilarin and altitudin A were synthesized by an in vitro cell-free protein synthesis (IV-CFPS) protocol coupled to a split-intein-mediated ligation (SIML) procedure for ligation of peptides and proteins [18]. This experimental approach has been developed in our lab for the synthesis and production of previously described or putative circular bacteriocins, without the need of genes encoding a peptide or protein involved in their N- to C-terminal covalent link [18]. The results obtained in this work clearly show that the IV-CFPS protocol, coupled to the SIML procedure, allowed the in vitro production of pumilarin and altitudin A with antimicrobial activity against *P. damnosus* CECT 4797 as the indicator strain (Figure 4). However, no antimicrobial activity was observed with the IV-CFPS-produced CLB (bacteriocin 574-like), thus suggesting that either this peptide had no antimicrobial activity against the indicators tested, or that the peptide holds a different biological function. It also may happen that the CLB belongs to the class I group of ribosomally synthesized and post-translationally modified peptides (RiPPs) that undergo enzymatic modifications during their biosynthesis. However, the presence of genes encoding hypothetical proteins for the maturation and processing of CLB seem to be absent in *B. altitudinis* ECC22.

Most importantly, the synthesis, production and secretion of pumilarin and altitudin A was confirmed in the supernatants of *B. altitudinis* EC222 using a multiple-step chromatographic procedure including two rounds of purification by RP-FPLC, followed by MALDI-TOF MS analysis of the eluted fractions with antimicrobial activity. The results obtained (Figure 5) suggest the presence in the eluted fraction 12 of a major peptide fragment of 6599.20 Da corresponding to the deduced molecular mass of altitudin A, and the presence in the eluted fraction 15 of two major peptide fragments of 6598.93 Da and 7089.15 Da, which corresponds to the deduced molecular mass of altitudin A and pumilarin, respectively. Thus, the purification of these bacteriocins from the supernatants of *B. altitudinis* ECC22 was not achieved to homogeneity. 

The confirmation of the circularization of pumilarin and altitudin A, secreted by *B. altitudinis* ECC22, was determined by trypsin digestion of the RP-FPLC-purified peptide fragments with antimicrobial activity, further subjected to LC-MS/MS-derived proteomics identification and combined with massive peptide analysis of the trypsin-derived fragments (Table 1). The results obtained allowed the identification, in the RP-FPLC-purified fractions, of peptide fragments containing the amino acids WL linked together, thus confirming the native head-to-tail covalent link of both circular bacteriocins in the purified supernatants of *B. altitudinis* ECC22 (Table 1). 

In both circular bacteriocins, the circularization event occurs between an L in the N- and a W in the C-terminal amino acid residue of the mature bacteriocins (Figure 4). This seems to be a common feature among class I circular bacteriocins which are known to present an L, V or W in their N-terminus, all hydrophobic amino residues of similar size (117–149 Da) and a W, Y or F amino acid residue in their C-terminus [32]. The presence of hydrophobic residues in the ligation site seems to be essential for the interaction with their cognate biosynthetic enzymes. Substitution of L1 in enterocin NKR-5-3B by other hydrophobic residues prone to promote helical structures (A, I, V and F) successfully yielded the mature bacteriocin, while substitutions of L1 by non-helix-promoting amino acid residues and non-hydrophobic residues failed to yield the mature bacteriocin [25].

To evaluate the antimicrobial effect and spectrum of activity of pumilarin and altitudin A as separate bacteriocins, and because of the difficulties encountered in their purification to homogeneity from the supernatants of *B. altitudinis* ECC22, we undertook their in vivo production and purification in from recombinant *E. coli* BL21 (DE3) (pCir-Pum) and *E. coli* BL21 (DE3) (pCir-Alt) heterologous producers of pumilarin and altitudin A, respectively. This protocol has been previously optimized and validated by our group for the in vivo production and purification of the circular bacteriocin garvicin ML [18]. Upon induction of the cultures with IPTG and sonication of the producer cells, the presence of pumilarin and altitudin A in the hydrophobic-interaction chromatography-derived eluted fractions was confirmed by MALDI-TOF MS. Thus, the in vivo heterologous production of circular bacteriocins by recombinant *E. coli* cells transformed with pUC-derived vectors driving the synthesis of circular bacteriocins under the control of a SIML procedure for ligation of peptides and proteins [18] opens the door for the future production and characterization of many other, not yet characterized, putative circular bacteriocins. 

The evaluation of the antimicrobial activity of pumilarin and altitudin A, produced by the recombinant *E. coli* BL21 (DE3) cells, against different bacterial indicator strains revealed a narrow spectrum of activity, being mostly active against other *Bacillus* spp. and *Listeria* spp. (Table 2). However, pumilarin was also active against other Gram-positive bacteria such as *L. lactis* and *S. suis*, but not against *E. coli* DH5α. These differences in their spectrum of activity suggest differences in their mode of action, although this observation has yet to be further evaluated.

The 3D structures predicted for pumilarin and altitudin A revealed that they hold five and four α-helices, respectively, and that their circularization site is located within a helical structure which contains mostly hydrophobic residues (Figure 6). It has been proposed that hydrophobic residues, close to the circularization site, may play an important role in the interaction between linear peptides and their circularization by bringing both N- and C-terminal ends in close proximity, allowing cyclisation to take place [23,32]. The homology models generated by the Robetta server are consistent with the results obtained with other closely related bacteriocins, such as enterocin AS-48 and enterocin NKR-5-3B, whose 3D structures have been experimentally elucidated [30,38]. Most evaluated circular bacteriocins are known to adopt a common 3D structure of four to five α-helices folded into a globular bundle enclosing a hydrophobic core [32,39,40]. 

## 4. Materials and Methods

### 4.1. Isolation and Screening of Bacterial Isolates with Antimicrobial Activity from Soil-Derived Samples

Bacterial isolates with antimicrobial activity were identified and isolated from soil-derived samples by a screening procedure carried out by 30 students from the “Centro de Educación Infantil, Primaria y Secundaria Obligatoria” (CEIPSO) El Cantizal (Las Rozas, Madrid) during their participation in the Service-Learning Project Micromundo in March 2022. The activity took place during three days (March 9th, 12th and 17th). The screening procedure (Figure S1) was adapted to their age and shared classrooms and, accordingly, the selection of the bacterial isolates was done under non-sterile conditions. Briefly, soil-derived samples from different places and locations were collected by the students, and 1 g of sample was diluted in 9 mL of a 0.85% NaCl (Scharlab, Barcelona, Spain) sterile saline solution, homogenized by vigorous shaking, and serially 10-fold diluted in the same sterile saline solution. Then, 0.1 mL of each dilution was poured into agar plates with Tryptic Soy Broth (TSB) (Oxoid Ltd., Basingstoke, UK) supplemented with 1,5% (*w*/*v*) of agar (Scharlab, Barcelona, Spain). Plates were then incubated at 37 °C for 5 days until colonies were observed. Bacterial isolates were then handpicked with a sterile toothpick and transferred into the brain heart infusion (BHI) agar plates (Oxoid) previously inoculated using a sterile swab (Deltalab, Barcelona, Spain), with the indicator strains *Kocuria rhizophila* CECT 241 and *Acinetobacter baylyi* ATCC 33305, and with their inoculums adjusted to 0.5 McFarland scale with a sterile saline solution. The plates were then incubated at 37 °C for 24 h, until zones of inhibition were observed.

### 4.2. Bacterial Strains, Media and Growth Conditions

*Bacillus altitudinis* ECC22 was grown in (BHI) broth (Oxoid) at 37 °C in agitation at 250 rpm in an orbital shaker (Ecotron, Infors HT, Braunschweig, Germany). *Escherichia coli* DH5α and *E. coli* BL21 (DE3) were grown in Luria Bertani (LB) broth (Scharlab) at 37 °C in agitation at 250 rpm and, when required, ampicillin (Sigma-Aldrich, Inc., St. Louis, MO, USA) was added to the cultures at 100 μg/mL. The *Lactococcus lactis* strains were grown in M17 broth (Thermo Fisher Scientific, Waltham, MA, USA), supplemented with 0.5% (wt/vol) glucose (GM17), and incubated at 32 °C without shaking. *Pediococcus damnosus* CECT 4797, *Ligilactobacillus salivarius* P1CEA3, *Enterococcus faecalis* 721 and *E. faecium* PE7 were grown in de Man, Rogosa, and Sharpe (MRS) broth (Oxoid) at 32 °C without agitation. The rest of the indicator strains, including *Bacillus safensis* LTh12, *B. pumilus* PE12, *B. cereus* CM7, *B. cereus* ICM17/00252, *B. thuringensis* CM4, *B. toyonensis* MG3, *B. toyonensis* NM11, *Listeria monocytogenes* CECT 4032, *L. seeligeri* CECT 917, *Staphylococcus aureus* 4, *S. aureus* ZTA11/00117ST, *Streptococcus agalactiae* DICM11/00863 and *S. suis* CECT 958, were grown in BHI broth (Oxoid) at 37 °C without agitation, except *Clostridium perfringens* DICM15/00067-5A, which was grown in anaerobic jars with AnaeroGen 3.5L sachets (Oxoid). Agar plates were prepared by adding 1.5% (*w/v*) agar (Scharlab).

### 4.3. Taxonomic Identification and Whole Genome Sequencing of Bacillus altitudinis ECC22

For the initial taxonomic identification of *B. altitudinis* ECC22, the InstaGene^TM^ matrix (BioRad, Hercules, CA, USA) resin was used for extraction and purification of the genomic DNA. The isolated DNA was further used as a template to amplify a variable region of the 16S rRNA gene, using primers rD1 (5′-TAAGGAGGTGATCCAGCC-3′) and fD1 (5′-AGAGTTTGATCCTGGCTCAG-3′) (Weiberg et al., 1991). The PCR product was purified with the NucleoSpin^R^ Gel and PCR Clean-up columns (Macherey-Nagel, Düren, Nordhein-Westfalen, Germany), and subjected to Sanger sequencing (Eurofins Genomics, Ebersberg, Germany). To determine the most probable identity of the strain, a comparative sequence analysis (BLASTn) was performed against the available sequence data in the National Center for Biotechnology Information (NCBI) database.

The total genomic DNA of *B. altitudinis* ECC22 was also obtained using the DNeasy Blood & Tissue Kit (Qiagen, Hilden, Germany). Purified DNA was quantified in a Qubit fluorometer (Invitrogen, Thermo Fisher Scientific) and its quality confirmed by agarose gel electrophoresis in 0.8% (*w/v*) agarose gels (Condalab, Madrid, Spain), visualized with a ChemiDoc Imaging System (Bio-Rad). The whole genome sequencing of the purified DNA was performed by Illumina and Oxford Nanopore Technologies (ONT) at the SeqCenter (Pittsburgh, PA, USA). For Illumina sequencing, all libraries were prepared using the tagmentation-based and PCR-based Illumina DNA Prep kit (Illumina, San Diego, CA, USA) and custom IDT 10 bp unique dual indices (UDI) with a target insert size of 280 bp. These libraries were sequenced on an Illumina NovaSeq 6000, producing paired-end 2 × 151 bp reads. For Nanopore sequencing, sample libraries were prepared using the Oxford Nanopore Technologies (ONT) Native Barcoding Kit 24 V14 (SQK-NBD114.24) (ONT, Oxford, UK), to manufacturer’s specifications. These libraries were sequenced on an ONT R10.4.1 flow cell on a GridION (ONT). Sequencing quality and adapter trimming was performed with bcl2fastq v.2.20.0.445 and porechop v.0.2.3_seqan2.1.1 for Illumina and ONT sequencing, respectively. The read count statistics were recorded. The hybrid assembly with Illumina and ONT reads was performed with Unicycler v.0.4.8 [41]. The quality of the assembled sequences was assessed using the QUAST v.5.0.2 tool [42]. The resulting DNA sequences were obtained in FASTA format. Unless otherwise stated, the bioinformatics analyses were performed from the assembled genome FASTA sequence file as the input file. The bacterial species identification was confirmed by KmerFinder v.3.0.2 (https://cge.cbs.dtu.dk/services/KmerFinder/, accessed on 8 January 2024), which predicts bacterial species using a K-mer algorithm. The annotation of the genome was performed with the Rapid Annotation Subsystem Technology (RAST) online server (http://rast.nmpdr.org/, accessed on 8 January 2024). For bacteriocin and ribosomally synthesized and post-translationally modified peptides (RiPPs) mining, the assembled genome was analyzed under default settings in the online webserver BAGEL v.4.0 (http://bagel4.molgenrug.nl/, accessed on 8 January 2024) [43] and the Antibiotics and Secondary Metabolite Analysis Shell (AntiSMASH) (https://antismash.secondarymetabolites.org/, accessed on 8 January 2024) [44]. The SnapGene 6.2.1. software (GSL Biotech, San Diego, CA, USA) was used for the analysis of the bacteriocin operons. BLASTp (NCBI) and UniProt were used to confirm peptide and protein sequences of the bacteriocins identified.

### 4.4. In Vitro Cell-Free Protein Synthesis (IV-CFPS) Coupled to a Split-Intein-Mediated Ligation (SIML) Procedure for the Synthesis, Production and Determination of the Antimicrobial Activity of Pumilarin and Altitudin A

The plasmids pCirc-Pum and pCirl-Alt were used as templates for the in vitro cell-free protein synthesis (IV-CFPS) and split-intein-mediated ligation (SIML) of the putative circular bacteriocins pumilarin and altitudin A, respectively. The design of both plasmids was based in an experimental procedure previously described by our research group [18]. Briefly, two synthetic gene constructs containing the C-and N-terminal fragments from the *Nostoc puntiforme* (Npu) DnaE split-intein, flanking the gene coding the mature peptide of both bacteriocins under study, were designed. For pumilarin, S37 was selected as the first amino acid residue in the linear conformation (S1 in the new peptide) and G36 as the last residue (G70 in the new peptide). For altitudin A, S8 was selected as the first amino acid residue in the linear conformation (S1 in the new peptide) and I7 as the last residue (I65 in the new peptide). In addition, and to reduce the toxicity of the intein peptides in *E. coli*, a protein degradation tag (SsrA) was included in the C-terminus of the gene constructs (Figure 4). Once the linear amino acid sequences of the constructs were designed, they were reverse-translated according to the codon usage of *E. coli* (www.bioinformatics.org/sms2/rev_trans.html, accessed on 8 January 2024) and placed under the control of a pUC-derived expression vector containing a T7 promoter region, a start codon (ATG), a stop codon (TAA) and a T7 terminator region. The designed gene constructs in the pUC-derived vectors (pCirc-Pum and pCir-AltA, respectively) were obtained from GeneArt (Life Technologies/Thermo Fisher Scientific).

The gene encoding of the predicted mature sequence of the closticin 574-like bacteriocin (*clb*) was PCR-amplified. A forward primer containing the T7 promoter sequence followed by the first 24 nucleotides of mature *clb* (5-GCGAATTAATACGACTCACTATAGGGCTTAAGTATAAGGAGGAATATGCCAGATTGGTCTAAGATCGCTGCA-3′) and a reverse primer containing the T7 terminator nucleotide sequence followed by the last 24 nucleotides of the mature *clb* (5-AAACCCCTCCGTTTAGAGAGGGGTTATGCTAGTTACCAATTTATCAAAGGCTAGGCC-3′) were synthesized. The oligonucleotide primers were obtained from Thermo Fisher Scientific. PCR amplifications were performed with the Phusion Hot Start II High-Fidelity DNA Polymerase (Thermo Fisher Scientific) in 50 µL reaction mixtures containing 1 µL of purified DNA. PCR reactions were visualized by agarose gel electrophoresis in a ChemiDoc Imaging System (Bio-Rad) and quantified in a Qubit fluorometer (Invitrogen, Thermo Fisher Scientific).

Plasmids pCirc-Pum and pCirc-AltA served as templates for the IV-CFPS protocol coupled to the SIML procedure, driving the in vitro synthesis and production of pumilarin and altitudin A, respectively, by using the PURExpress In Vitro Protein Synthesis Kit (New England Biolabs, Ipswich, MA, USA), as previously described [18]. In all cases, the DNA templates were used at a final concentration of 10 ng/µL in 25 µL reactions, maintained at 37 °C for 2 h, and then placed on ice to stop the reaction. The antimicrobial activity of the IV-CFPS/SIML reactions was evaluated by using a spot-on-agar test (SOAT) [45]. Briefly, 5 μL samples of the IV-CFPS/SIML reactions were deposited on the surface of Petri plates overlaid with a soft-agar (0.8%) culture of the indicator microorganism (ca. 10^5^ cfu/mL) *Pediococcus damnosus* CECT 4797. The plates were then incubated at 37 °C for 24 h, until zones of inhibition were observed.

However, the PCR-derived amplicon containing the mature *clb* served as a template for the IV-CFPS synthesis and production of the putative bacteriocin CLB by using the same PURExpress In Vitro Protein Synthesis Kit (New England Biolabs), as previously described [18]. The DNA templates were also used at a final concentration of 10 ng/µL in 25 µL reactions, maintained at 37 °C for 2 h, and then placed on ice to stop the reaction. The antimicrobial activity of the IV-CFPS reactions was evaluated by using a SOAT, in which 5 μL samples of the IV-CFPS reactions were deposited on the surface of Petri plates overlaid with a soft-agar (0.8%) culture of the indicator microorganism (ca. 10^5^ cfu/mL) *Pediococcus damnosus* CECT 4797 and *C. perfringens* DICM15/00067-5A. The plates were then incubated at 37 °C for 24 h.

### 4.5. Purification of the Bacteriocins Produced by B. altitudinis ECC22

Bacteriocins were purified from 2 L cultures of *B. altitudinis* ECC22, grown at 32 °C for 24 h in BHI broth under constant agitation (250 rpm) and centrifuged at 8000 rpm for 10 min at 4 °C to obtain the corresponding cell-free supernatants (CFS). Then, the CFS were subjected to precipitation with ammonium sulphate (Sigma-Aldrich), desalted by gel filtration (PD-10 columns from Cytiva, Marlborough, MA, USA), and further subjected to hydrophobic-interaction (Octyl-Sepharose CL-4B, Cytiva) chromatography, followed by two rounds of reverse-phase chromatography in an ÄKTA purifier fast protein liquid chromatography (RP-FPLC) system (GE Healthcare Life Sciences, Barcelona, Spain). For the first RP-FPLC round of purification, the sample was applied into a SOURCE 5RPC ST 4.6/150 column (GE Healthcare Life Sciences) and the retained compounds eluted with a gradient of 0% to 100% isopropanol (Thermo Fisher Scientific) with 0.1% (*v*/*v*) trifluoroacetic acid (TFA) (Panreac, Madrid, Spain). The eluted fractions were monitored at 254 nm (A_254_), filtered through 0.22 μm filters (Sartorius, Göttingen, Germany), and their antimicrobial activity quantified by a microtiter plate assay (MPA) against *P. damnosus* CECT 4749 as the indicator microorganism [13,18]. For the MPA, the growth inhibition of sensitive cultures was measured spectrophotometrically at 620 nm with a FLUOstar OPTIMA (BMGLabtech, Ortenberg, Germany) plate reader. One bacteriocin unit (BU) was defined as the reciprocal of the highest dilution of the bacteriocin that caused a growth inhibition of 50% (50% of the turbidity of the control culture without bacteriocin). The fractions with the highest antimicrobial activity were combined and then subjected to a second round of RP-FPLC, as described above.

### 4.6. MALDI-TOF MS Analysis and LC-MS/MS-Derived Targeted Proteomics Identification Combined with Massive Peptide Analysis, of the Bacteriocins Produced by B. altitudinis ECC22

Purified CFS fractions from *B. altitudinis* ECC22, with the highest antimicrobial activity after their second round of RP-FPLC, were evaluated for determination of their molecular mass by Matrix-Assisted Laser Desorption/Ionization Time of Flight Spectrometry (MALDI-TOF MS) at the Unidad de Espectrometría de Masas (CAI Técnicas Químicas, UCM, Madrid, Spain). Briefly, 1 µL of samples was mixed with 1 µL of a sinapic acid matrix (Sigma-Aldrich) in 30% acetonitrile and 0.3% TFA (Panreac) and then applied directly to the MS target plate and dried under a stream of warm air. The analysis of samples was done on an Ultraflex workstation (Bruker Daltonics, Billerica, MA, USA) equipped with a 337 nm nitrogen laser. The mass spectrometer was calibrated with protein calibration standard I (4000–20,000 *m*/*z*) from Bruker Daltonics. The FlexControl Software v.2.4. (Bruker Daltonics) was used for sample analysis and control of method parameters.

Purified CFS fractions from *B. altitudinis* ECC22, with the highest antimicrobial activity after their second round of RP-FPLC, were also subjected to LC-MS/MS analysis at the Unidad de Proteómica (CAI Técnicas Biológicas, UCM, Madrid, Spain). For LC-MS/MS analysis, the peptides and proteins in the samples were digested with trypsin into S-Trap^TM^ micro columns (ProtiFI), following the manufacturer’s instructions (Roche Molecular Biochemicals, NJ, USA). Approximately 1 μg of the produced peptides was analyzed by liquid nano-chromatography in a Vanquish Neo (Thermo Fisher Scientific) coupled to a Q-Exactive HF high-resolution mass spectrometer (Thermo Fisher Scientific). The peptides were eluted using a 30 min gradient from 2% to 35% buffer B (0,1% formic acid [FA] in 2% acetonitrile [ACN]) in Buffer A (0.1% FA in dH_2_O) at a constant low rate of 250 nl/min. Data acquisition in a Q-Exactive HF was performed using a combined targeted proteomics method (data-dependent acquisition plus an inclusion list) to detect the peptides of interest in the samples. The inclusion list with the *m*/*z* of fragments from the predicted digestion of the complete amino acid sequence of the peptides pumilarin and altitudin A was obtained with the free program Skyline v.20.2 (https://skyline.gs.washington.edu, accessed on 8 January 2024). The MS/MS spectra were acquired with a resolution of 30,000 in positive mode.

Peptide identifications from the MS/MS data were performed with the Proteome Discoverer 2.4 software (Thermo Fisher Scientific) using the MASCOT v 2.6 or Sequence HT search engines and Peaks Studio v 10.5 software (Bioinformatic solution Inc., Waterloo, ON, Canada) that has additional tools, such as de novo sequencing, to maximize the number of peptides and proteins identified. Database searches were performed against three databases: SwissProt downloaded from Uniprot (https://www.uniprot.org, accessed on 8 January 2024), Target DB database for the 2 target bacteriocins of *B. altitudinis* ECC22, and a Contaminants DB (www.matrixscience.com/help/seq_db_setup_contaminants.html, accessed on 8 January 2024) database.

### 4.7. In Vivo Production of Pumilarin and Altitudin A by Recombinant Escherichia coli BL21 (DE3)

Plasmids pCirc-Pum and pCirc-Alt were transformed into competent *E. coli* BL21 (DE3) cells, following the procedure recommended by the manufacturer (Thermo Fisher Scientific). Then, 1 L of Terrific Broth (containing 1.2% tryptone [Oxoid], 2.4% yeast extract [Fisher Scientific, Pittsburgh, PA, USA], 72 mM K_2_HPO_4_ [Fisher Scientific], 17 mM KH_2_PO_4_ [Fisher Scientific] and 0.4% glycerol [Fisher Scientific]), supplemented with ampicillin (Sigma-Aldrich) at a concentration of 100 μg/mL was seeded, until an OD_600_ of 0.1 with an overnight culture of *E. coli* BL21 (DE3) (pCirc-Pum) or *E. coli* BL21 (DE3) (pCirc-Alt), grown in LB-Amp. The cultures were maintained at 37 °C in agitation at 250 rpm and, when the grown cells reached an OD_600_ of approximately 0.4, the isopropyl-β-D-thiogalactoside (IPTG) (Thermo Fisher Scientific) inducer was added to a final concentration of 0.5 mM. The cultures were grown for another 3 h, and the cells pelleted by centrifugation at 8000× *g* for 15 min at 4 °C. The cells were resuspended in 20 mL ice-cold 20 mM phosphate buffer (all reagents from Fisher Scientifics), pH 6.0 with 1 M NaCl (Scharlab), and lysed by sonication (6 cycles of 10 s at 45% maximum intensity), with 1 min incubation in ice between cycles in a 450 Digital Sonifier (Branson Ultrasonics, Brookfield, CT, USA). The insoluble cell debris was pelleted by centrifugation at 8000× *g* for 15 min at 4 °C, and the resulting cellular soluble fraction (CSF) filtered through a 0.45 μm filter (Sartorius, Goettinggen, Germany). Ammonium sulfate (Sigma-Aldrich) (10% *w*/*v*) was added to the CSF, which was further deposited on a column with 2 mL of Octyl Sepharose CL-4B (GE Healthcare Life Sciences), previously washed with dH_2_O and equilibrated with 15 mL of 20 mM phosphate buffer, pH 6.0 with ammonium sulfate (1% *w*/*v*), named equilibration buffer (EB). The bacteriocins were eluted from the column with 10 mL 70% ethanol (Fisher Scientific) diluted in 20 mM phosphate buffer, pH 6.0. The presence of pumilarin and altitudin A in the eluted fractions with antimicrobial activity was confirmed by MALDI-TOF MS, as previously described.

### 4.8. Antimicrobial Activity of Pumilarin and Altitudin A, Produced by Recombinant Escherichia coli BL21 (DE3) against Different Indicator Strains

The antimicrobial activity of pumilarin and altitudin A, produced by *E. coli* BL21 (DE3) (pCir-Pum) and *E. coli* BL21 (DE3) (pCir-Alt), were evaluated by using a spot-on-agar test (SOAT), as described above. Briefly, eluted fractions with the highest antimicrobial activity from the purified CSF of the *E. coli* BL21 (DE3)-bacteriocin producers, recovered by interaction chromatography (Octyl Sepharose CL-4B column), were deposited on the surface of Petri plates overlaid with a soft-agar (0.8%) culture of different indicator bacteria (ca. 10^5^ cfu/mL). The antimicrobial activity of pumilarin and altitudin A was evaluated with both bacteriocins alone and combined. When tested alone, 2.5 µL of each bacteriocin was mixed with 2.5 µL of dH_2_O. When tested together, both bacteriocins were mixed at an equal ratio (2.5 µL: 2.5 µL).

### 4.9. Computational Modeling of the Three-Dimensional (3D) Structures of Pumilarin and Altitudin A

Prediction of the 3D structures of pumilarin and altitudin A was carried out using the Robetta bioinformatics (https://robetta.bakerlab.org/, accessed on 8 January 2024) server. The 3D models generated were evaluated further with the ChimeraX (https://www.cgl.ucsf.edu/chimerax/, accessed on 8 January 2024) server [46].

## 5. Conclusions

This study highlights the importance of searching and characterizing antimicrobial compounds produced by microorganisms from different environmental niches (e.g., bacteria from soil), different from traditional antibiotics, in the search for strategies to tackle antibiotic-resistant bacteria. While numerous studies often focus on the identification of gene clusters potentially encoding antimicrobial compounds, it becomes imperative to move beyond a mere genomic determination and experimentally evaluate the active expression of these genes, ensuring they genuinely encode bioactive compounds. The identification of putative bacteriocin gene clusters, and the subsequent functional characterization of pumilarin and altitudin A, showcase innovative methodologies that bridge bioinformatic analyses with experimental validation, offering insights into their synthesis, production, and antimicrobial activity. Further investigations into the structural properties and modes of action of these and other circular bacteriocins hold promise for advancing efforts in finding novel antimicrobial compounds against antibiotic-resistant pathogens, emphasizing the necessity to expand the repertoire of characterized bacteriocins and investigate their therapeutic potential.

## Figures and Tables

**Table 1:** Peptide fragments, identified by LC-MS/MS targeted proteomics combined with massive peptide analysis of the trypsinized fractions 12 and 15, obtained after the second RP-FPLC round of purification of the CFS of *B. altitudinis* ECC22.

RP-FPLC-Purified Fraction 12			
Peptide Sequence	Theoretical MH+ (Da)	Bacteriocin in Sample	Detected *m*/*z*
YAAEWLATNLGISR	1564.8118	Altitudin A	782.9106
AAEWLATNLGISR	1401.7484	Altitudin A	701.3768
AEWLATNLGISR	1330.7113	Altitudin A	665.8602
YAAEWLATNLGIS	1408.7107	Altitudin A	704.8593
ATNLGISR	831.4682	Altitudin A	416.2385
YAAEWLATNL	1151.5731	Altitudin A	576.2907
TNLGISR	760.4311	Altitudin A	380.7194
WLATNLGISR	1130.6316	Altitudin A	565.8204
YLKNEIK	907.5247	Pumilarin	454.2659
**RP-FPLC-Purified Fraction 15**			
**Peptide Sequence**	**Theoretical** **MH+ (Da)**	**Bacteriocin** **in Sample**	**Detected** ***m*/*z***
GLSLIAAAGK	900.5512	Pumilarin	450.7803
AVIAWLAK	871.5400	Pumilarin	436.2736
YLKNEIK	907.5247	Pumilarin	454.2661
YAAEWLATNLGISR	1564.8118	Altitudin A	782.9104
LATNLGISR	944.5523	Altitudin A	472.7805
TNLGISR	760.4311	Altitudin A	380.7193
GTGLVTAAMVATAK	1306.7035	Altitudin A	653.8569
ATNLGISR	831.4682	Altitudin A	416.2401
YAAEWLAT	924.4461	Altitudin A	462.7273
TGLVTAAMVATAK	1249.6820	Altitudin A	625.3439
LVTAAMVATAK	1091.6129	Altitudin A	546.3106
YAAEWLATNL	1151.5731	Altitudin A	576.2930

**Table 2:** Antimicrobial effect and spectrum of activity of pumilarin and altitudin A, produced by recombinant *E. coli* BL21 (DE3) cells, against different indicator strains.

		Antimicrobial Activity ^a^		
Indicator Strain	Origin ^b^	Pumilarin	Altitudin A	Pumilarin + Altitudin A
*Bacillus safensis* LTh12	DNBTA	++	++	++
*Bacillus pumilus* PE12	DNBTA	++	++	+++
*Bacillus cereus* CM7	DNBTA	+	+	+
*Bacillus cereus* ICM17/00252	VISAVET	++	+++	++
*Bacillus thuringiensis* CM14	DNBTA	-	++	+
*Bacillus thuringiensis* AP5	DNBTA	-	++	++
*Bacillus toyonensis* MG3	DNBTA	+	-	+
*Bacillus toyonensis* NM11	DNBTA	++	++	++
*Pediococcus damnosus* CECT 4797	CECT	+++	-	+++
*Listeria seeligeri* CECT 917	CECT	+++	++	+++
*Listeria monocytogenes* CECT 4032	CECT	+++	+++	+++
*Ligilactobacillus salivarius* P1CEA3	DNBTA	-	-	-
*Lactococcus lactis* NZ9000	NIZO	-	-	-
*Lactococcus lactis* LMGT3797	LMG	+++	-	+++
*Lactococcus lactis* LMGT3798	LMG	++	-	+++
*Enterococcus faecalis* 721	SM-HRC	-	-	-
*Enterococcus faecium* PE7	SM-HRC	-	-	-
*Staphylococcus aureus* ZTA11/00117ST	VISAVET	-	-	-
*Staphylococcus aureus* 4	SM-HRC	-	-	-
*Streptococcus suis* CECT 958	CECT	++	-	++
*Streptococcus agalactiae* DICM11/00863	VISAVET	-	-	-
*Escherichia coli* DH5α	DNBTA	-	-	-

^a^ Antimicrobial activity as the diameter of halos of inhibition: -: no inhibition, +: <5 mm, ++: 5–10 mm, +++: >10 mm. ^b^ VISAVET: Centro de Vigilancia Sanitaria Veterinaria, Universidad Complutense de Madrid (UCM) (Madrid, Spain). CECT: Colección Española de Cultivos Tipo (Valencia, Spain). DNBTA: Departamento de Nutrición, Bromatología y Tecnología de los Alimentos, Facultad de Veterinaria, Universidad Complutense de Madrid (UCM) (Madrid, (Spain). NIZO—Department of Biophysical Chemistry, NIZO Food Research (Ede, The Netherlands). LMG: Laboratory of Microbial Gene Technology, Norwegian University of Life Sciences (Ås, Norway). SM-HRC: Servicio de Microbiología, Hospital Universitario Ramón y Cajal (Madrid, Spain).

## Data Availability

The whole genome assembly of *B. altitudinis* ECC22 is deposited in NCBI under the Bioproject accession number PRJNA1035429.

## Supplementary Materials

The following supporting information can be downloaded at: https://www.mdpi.com/article/10.3390/ijms25042020/s1.
