# Supplementary material for: Production of Pumilarin and a Novel Circular Bacteriocin, Altitudin A, by Bacillus altitudinis ECC22, a Soil-Derived Bacteriocin Producer
Source: Int J Mol Sci. 2024 Feb 7;25(4):2020. doi: 10.3390/ijms25042020 (PMC10888436; doi:10.3390/ijms25042020)
Supplement: Supplementary file 1 [file ijms-25-02020-s001.zip › ijms-2840732-supplementary.pdf]

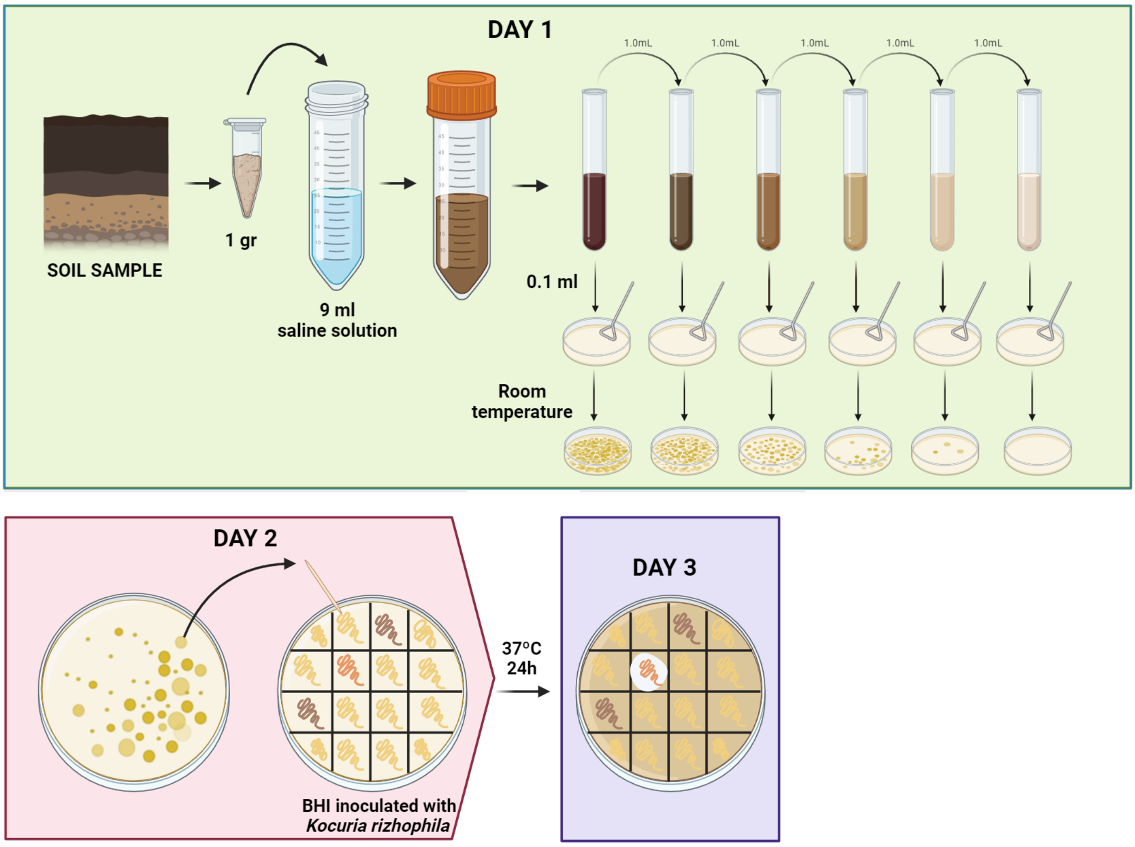

Figure S1. Schematic view of the screening workflow carried out by students from the CEIPSO El Cantizal.

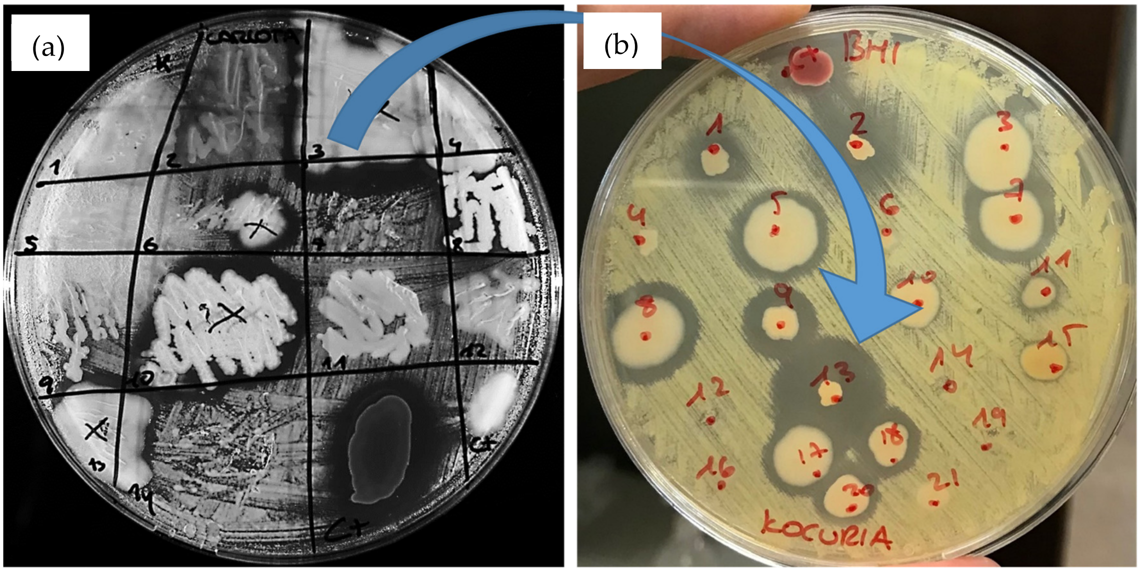

Figure S2. Direct antimicrobial activity of soil-derived bacterial isolates. (a) A number of colonies isolated from soil were tested against the gram positive indicator *Kocuria rhizophila* CECT 24, by students from the CEIPSO El Cantizal. Colony 3 was selected for its large antimicrobial activity. (b) Confirmation of the antimicrobial activity of the most active isolates against *Kocuria rhizophila* CECT 241. Colony 13, corresponding to colony 3 in plate (a), was selected for its large antimicrobial activity.

Table S1. Assembly quality assessment of *B. altitudinis* ECC22 genome using Quast.

	Assembly
# contigs (>= 0 bp)	1
# contigs (>= 1000 bp)	1
# contigs (>= 5000 bp)	1
# contigs (>= 10000 bp)	1
# contigs (>= 25000 bp)	1
# contigs (>= 50000 bp)	1
Total length (>= 0 bp)	3807059
Total length (>= 1000 bp)	3807059
Total length (>= 5000 bp)	3807059
Total length (>= 10000 bp)	3807059
Total length (>= 25000 bp)	3807059
Total length (>= 50000 bp)	3807059
# contigs	1
Largest contig	3807059
Total length	3807059
GC (%)	41.29
N50	3807059
N75	3807059
L50	1
L75	1
# N's per 100 kbp	0.00

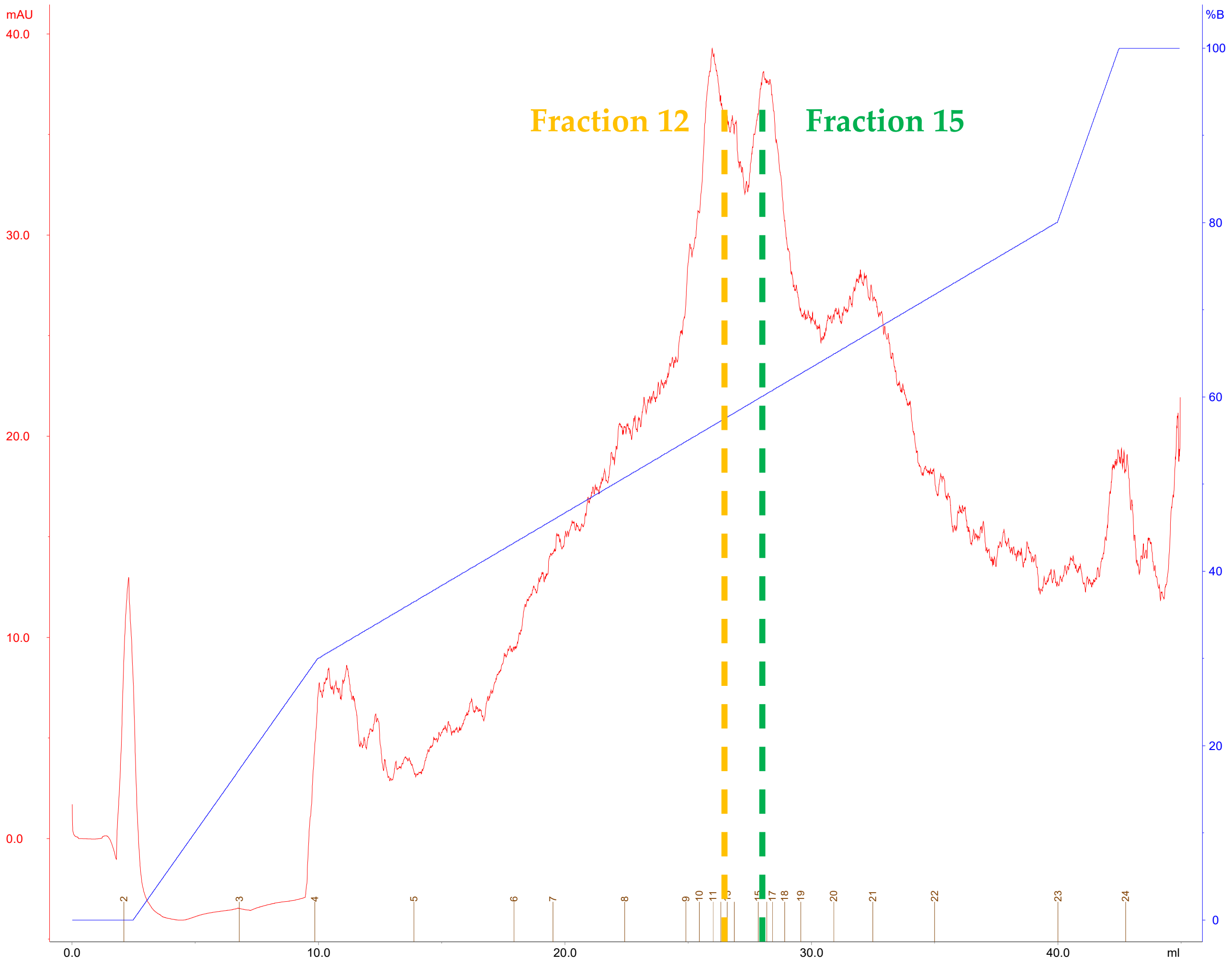

Figure S3. Elution profile after the second RP-FPLC round of purification of the CFS of *B. altitudinis* ECC22. Only the eluted fractions 12 (in yellow) and 15 (in green), showed antimicrobial activity against *P. damnosus* CECT 4797.
